# Efficacy of low-dose rituximab versus immunosuppressants in refractory orbital inflammatory pseudotumors with intracranial extension

**DOI:** 10.3389/fimmu.2025.1516909

**Published:** 2025-01-24

**Authors:** Yuyu Li, Mingming Sun, Xintong Xu, Biyue Chen, Xiyun Chen, Yuhang Wang, Quangang Xu, Huanfen Zhou, Shihui Wei

**Affiliations:** Senior Department of Ophthalmology, the Third Medical Center of Chinese People’s Liberation Army General Hospital & Chinese People’s Liberation Army Medical School, Beijing, China

**Keywords:** orbital inflammatory pseudotumors, inflammation with intracranial extension, treatment, rituximab, immunosuppressants

## Abstract

**Objective:**

The aim of this study was to compare the efficacy of low-dose rituximab (RTX) and immunosuppressants in treating orbital inflammatory pseudotumor (OIP) with intracranial extension, a refractory and high-relapse disease.

**Methods:**

Patients who had been diagnosed with refractory OIP with intracranial extension and who were refractory to systemic corticosteroids were retrospectively recruited at the Neuro-Ophthalmology Department at the Chinese People’s Liberation Army General Hospital between December 2018 and September 2022. After methylprednisolone pulse therapy, we added 2 mg of tacrolimus per day, 1500 mg of mycophenolate mofetil per day, or 200 mg of rituximab at days 1 and 15, and then monitored those with CD19+ B cells of under 1% as adjuvant therapy.

**Results:**

Eleven patients (six males and five females) were included, with a mean age of 45.5 ± 11.8 years (age range: 21–64 years). The average follow-up period was 3.8 years (range: 2–5). Eight patients (72.7%) had different levels of decreased vision at onset of the illness and four patients (36.4%) had severely impaired vision (three with no light perception, one with some light perception). Four patients (36.4%) showed clinical course worsening or lack of remission when treated with corticosteroids. Seven patients (63.6%) had a typical relapsing course, and the annual recurrence rate was higher than 7.36 ± 3.73 times. Of these seven, four (57.1%, 4/7) were able to undergo successful management with immunosuppressants. Three (42.9%, 3/7) failed with immunosuppressants but succeeded in controlling relapse with RTX.

**Conclusion:**

OIP with intracranial extension is uncommon. More than half of patients with OIP with intracranial extension may be satisfactorily treated with corticosteroids combined with immunosuppressants. However, for patients who still experience recurrence or slow reduction of lesions after applying this combined therapy, RTX may be a better option.

## Introduction

Originally known as orbital pseudotumor, orbital inflammatory pseudotumor (OIP) refers to a nonspecific acute inflammatory lesion affecting any tissue within the orbit. The first case of OIP was described in 1903 by Busse and Hochheim and later in 1905 by Birch-Hirschfeld, but the earliest report of OIP with intracranial extension was published in 1958 by H Jackson (1). It refers to inflammation through the superior orbital fissure, along the meninges into the cavernous sinus. This form of OIP is relatively uncommon; one study in 1992 showed that there was computerized tomography evidence of intracranial extension in 8.8% (8/90) of patients with OIP (2). At present, there are limited reports describing OIP (2–5). Because the lesion extends the superior orbital fissure to the cavernous sinus, cases of OIP intracranial extension may involve symptoms and signs of optic perineuritis, orbital apex syndrome and cavernous sinus syndrome causing severe vision impairment and multiple cranial nerve palsies. It is critical, therefore, to perform early and effective treatment.

OIP showed a dramatic response to appropriated doses of corticosteroids, with approximately three-quarters of patients showing marked improvement within 48 hours (6). However, relapse is common. Studies have shown that approximately 78% of patients treated with corticosteroids alone would have a relapse course, whereas this proportion decreases to 16% if combining with immunosuppressants (7, 8). In cases with extraorbital extension, corticosteroid therapy alone may not produce good results; combining with immunosuppressants or biologic therapy may be considered (3, 4, 9).

Tacrolimus and mycophenolate mofetil were developed more recently than other immunosuppressant agents for OIP. Tacrolimus is known to have excellent safety and efficacy (10). It has selective inhibitory effects on T-cell activation and T-cell-dependent B-cell activation, which provides an immunological basis for immunosuppressive treatment (10). It has shown good efficacy in 3 cases of OIP (5). Mycophenolate mofetil suppresses the immune system by reversibly inhibiting inosine-5-monophosphate dehydrogenase, resulting in a selective inhibition of replication of T and B lymphocytes. Studies have shown that mycophenolate mofetil may be an effective corticosteroid-sparing agent in the treatment of inflammatory eye disease, including OIP (11–13). However, about one-third of patients with OIP fail to respond optimally to immunosuppressive therapy (9). RTX is a monoclonal antibody that recognizes CD20, an antigen expressed on the surface of mature B-lymphocytes. B-lymphocytes are the progenitors of plasma cells; it is, therefore, reasonable to argue that there is a strong rationale for the use of RTX in OIP. The use of RTX has been reported with increasing frequency and with generally good efficacy in cases of refractory non-infectious/non-malignant orbital inflammation (14, 15). However, clinical characteristics and treatment of OIP intracranial extension need to be researched further. Here, we report 11 patients with OIP intracranial extension treated with tacrolimus, mycophenolate mofetil, or RTX as adjuvant therapy. We compared therapeutic effects between them to find a highly efficient method to control this refractory disease.

## Methods

### Study subjects

This observational study included 11 patients who had OIP that extended beyond the orbit to the intracranial. Patients were retrospectively recruited from the Department of Neuro-ophthalmology at the People’s Liberation Army General Hospital (PLAGH) in China from December 2018 to September 2022. The study protocol was approved by the Ethics Committee for Human Research at PLAGH (approval number S2023-106-02) and was in accordance with the tenets of the Helsinki Declaration and the ICH-GCP guidelines. Written informed consent was obtained from all the study subjects.

### Examination evaluation and laboratory tests

Diagnosis of OIP that extends beyond the orbit is based on clinical indicators, magnetic resonance imaging (MRI), selected normal laboratory findings and incisional biopsy (16). Two patients with atypical OIP underwent biopsies. IgG4-related disease, thyroid-associated ophthalmopathy, granulomatosis with polyangiitis and Crohn’s disease were excluded in all cases. All patients underwent orbital MRI with gadolinium-enhanced fat suppression sequence and complete ophthalmic examination, including best-corrected visual acuity (BCVA), intraocular pressure, visual field and optic coherence tomography (OCT). The laboratory evaluation of OIP included routine biochemistry, inflammatory markers and a comprehensive autoimmune panel: white blood cell count with differential, platelet count, calcium, liver function tests, erythrocyte sedimentation rate, C-reactive protein, serum IgG4, anti-Ro, anti-La, rheumatoid factor, antinuclear antibody titer, anti-neutroplasmic antibody, triiodothyronine, thyroxine, thyroid-stimulating hormone and thyroid-stimulating hormone receptor antibody. Lumbar puncture was performed in all patients, and white cell counts, total protein levels and concentrations of IgG in cerebrospinal fluid (CSF) were tested.

### Treatment protocol in OIP with intracranial extension

Patients were treated with intravenous methylprednisolone (started with 1,000 mg for three days) followed by oral prednisone (1 mg/kg/day), which was gradually tapered off over 12 weeks. Either 2 mg of tacrolimus or 1500 mg of mycophenolate mofetil were added per day, or 200 mg infusions of RTX were administered at days 1 and 15. This was followed by monitoring CD19+ B cells (RTX drug levels) of under 1%; repeat infusions were performed when if this level was close to 1%. All patients were followed up for at least one year.

### Definition of clinical endpoint

The primary efficacy endpoint was defined as being when eye symptoms alleviate and when the patient does not relapse during the two years of follow-up treatment using the immunosuppressant and RTX. During this time, patients were able to stop prednisone treatment successfully. Secondary efficacy criteria were that the lesions shown by MRI became smaller or disappeared.

## Results

### Clinical characteristics of patients with OIP with intracranial extension

Demographic and clinical characteristics information for enrolled patients is summarized in **Table 1**. The study cohort included eleven patients with OIP, consisting of six males and five females, with a mean age of 45.5 ± 11.8 years (age range: 21–64 years). Two patients (18.2%) had bilateral involvement. Ocular pain at disease onset was present in 81.8% (9/11) of the patients. Headache and diplopia were experienced by 27.3% (3/11) and 36.4% (4/11) of the patients, respectively. One patient (patient 2; 9.1%) had optic disk edema and showed retinal nerve fiber layer thickening and gradual thinning. This patient also showed vision field defects during the attack; these changed to normal during follow-up. Except for this patient, all patients had a normal fundus and normal retinal nerve fiber layer thickening. Seven patients (63.6%) had decreased vision at onset of illness; among them, four patients (57.1%, 4/7) had profound and permanently impaired vision (three with light perception, one with some light perception), and no single patient’s vision eventually improved. Two patients with profound vision impairment underwent biopsies to confirm the diagnosis and to rule out other orbital diseases, such as lymphoma and metastatic tumors.

**Table 1:** Demographic and clinical characteristics information for enrolled patients.

Patients/Sex/Age	Invloved eyes	Vision loss	VA at onset		Ocular pain	Headache	Diaplopia	Optic disk edema	Follow up VA	
			OD	OS					OD	OS
1/M/40	R	No	20/20	20/20	Yes	Yes	Yes	No	20/20	20/20
2/F/57	Bilateral	Yes	20/30	20/30	Yes	Yes	Yes	Yes	20/25	20/25
3/F/33	R	No	20/25	20/25	Yes	No	No	No	20/25	20/25
4/M/64	Bilateral	Yes	NLP	20/100	Yes	No	No	No	NLP	20/40
5/F/57	R	Yes	20/40	20/20	No	No	No	Yes	20/20	20/20
6/M/21	R	No	20/20	20/20	Yes	Yes	Yes	No	20/20	20/20
8/M/50	L	Yes	20/25	NLP	Yes	No	No	No	20/25	NLP
8/F/42	L	No	20/20	20/20	Yes	Yes	Yes	No	20/20	20/20
9/M/41	L	Yes	20/20	NLP	No	No	No	No	20/20	NLP
10/F/54	R	Yes	LP	20/25	Yes	No	Yes	No	LP	20/25
11/M/41	R	Yes	20/20	20/20	Yes	No	No	No	20/20	20/20

M, male; F, female; BCVA, best corrected visual acuity; OD, right laterality; OS, left laterality; NLP, no light perception; LP, light perception.

There was no obvious autoimmune antibody abnormality in any of the patients, and serum IgG4 and thyroid function were normal in all patients. Five patients (45.5%) had elevated total protein concentrations of 431.2 ± 30.8 mg/L (normal range: 150–400 mg/L) in the CSF. Two patients (18.2%) had elevated IgG concentrations in the CSF of 13.9 and 3.63 mg/dL (patients 2 and 6, respectively; normal range: 0–3.4 mg/L). CSF white blood cell counts were normal, and no further laboratory abnormalities, such as infection, were identified. An orbit MRI scan after contrast revealed unilateral or bilateral enhancement of the extraocular muscle and a lesion extending from the superior orbital fissure to the cavernous sinus. Patient 5 also showed enhancement in the perioptic nerve.

### Course and therapy in patients with OIP with intracranial extension

**Figure 1:**
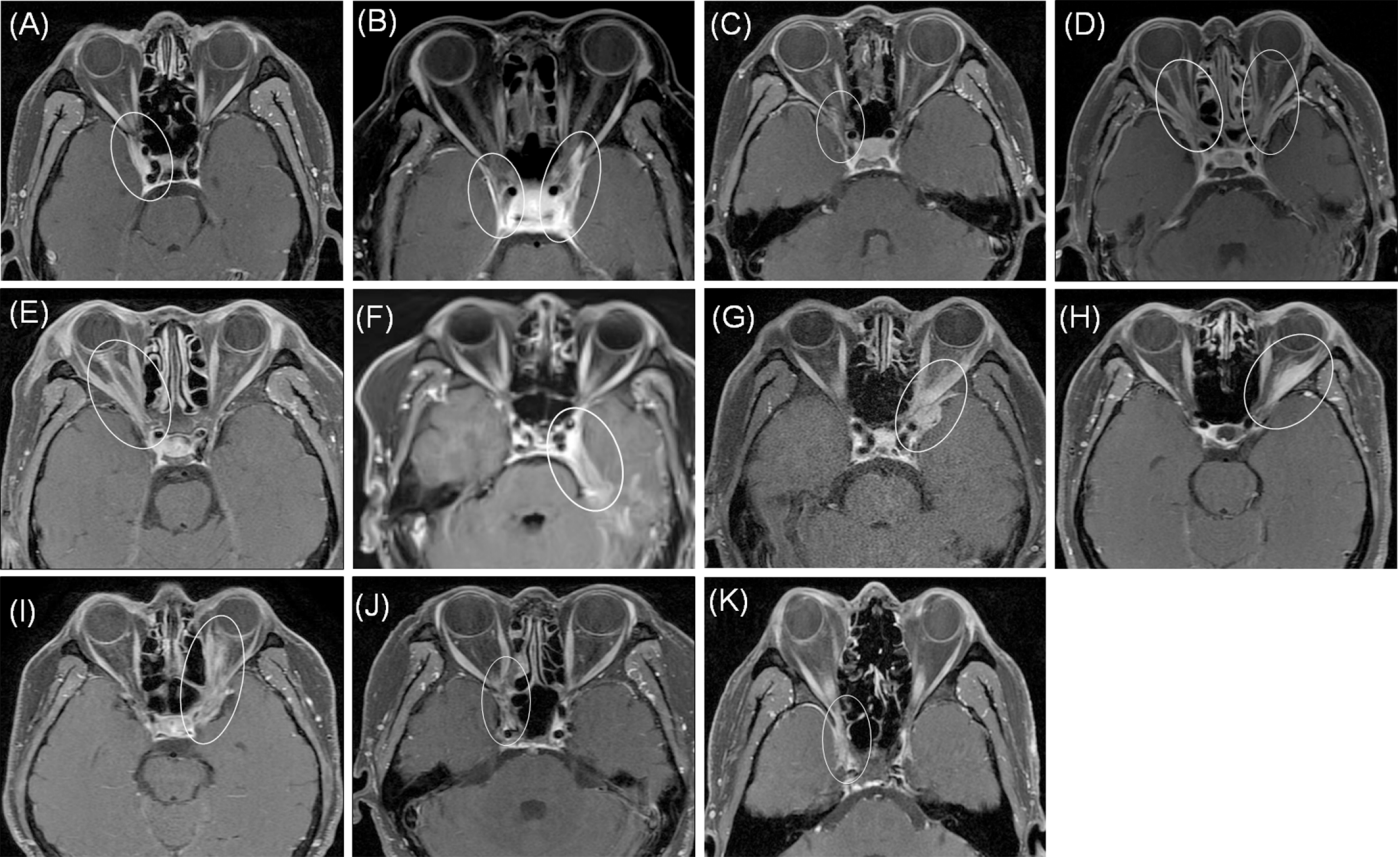
MRI axial showed abnormal enhancement of lesions (circle) from patient 1 to 11 **(A–K)**. Patients 4, 5 and 9 **(D, E, I)** showed enhancement of the optic nerve sheath. All patients showed involvement of cavernous sinus and dura.

**Figures 1A–K** shows patients’ orbit MRI scan results from patient 1 to 11. Patients 4, 5 and 9 had enhancement of the optic nerve sheath which can explain why vision acuity was decreased. **Table 2** shows the disease course evolution and therapy in patients with OIP with intracranial extension. Four patients (36.4%) showed clinical course worsening or lack of remission when treated with corticosteroids. Seven patients (63.6%) had a typical relapsing-remitting course, with a relapse course of two to more than seven times when treated with prednisone alone or with an immunosuppressant simultaneously, and the annual recurrence rate of the patients was higher than 7.36 ± 3.73 times (range: 3–12). They all showed a sensitive reaction to corticosteroids but had a relapse course when prednisone was in taper. Four patients (patients 1, 2, 3, 9) had a relapse course every time when prednisone was reduced to 15–40 mg.

**Table 2:** The clinical course and therapy in patients with OIP with intracranial extension.

Patients/Sex/Age	Duration at enrollment	Relapse times	ARR	Prednisone in relapse	Initial failure treatment	Revision Treatment	Follow-up time
1/M/40	3 month	3 times	12	40 mg	Pred, Tacrolimus	RTX	2.5 years
2/F/57	2 years	6 times	3	20 mg	Pred, Tacrolimus	RTX	2 years
3/F/33	2 years	>7 times	>3.5	15 mg	Pred, Tacrolimus	RTX	3 years
4/M/64	3 months	Worsening	/	/	Pred, MMF	RTX	5 years
5/F/57	2 years	Unremitting	/	/	Pred	Tacrolimus	2 years
6/M/21	6 months	>4 times	>8	0 mg	Pred	Tacrolimus	5 years
7/M/50	4 months	Unremitting	/	/	Pred	Tacrolimus	5 years
8/F/42	1.5years	6 times	8	0 mg	Pred	Tacrolimus	5 years
9/M/41	2 months	2 times	12	30 mg	Pred	MMF	3 years
10/F/54	3 months	Worsening	/	/	Pred	MMF	4 years
11/M/41	1 year	5 times	5	0 mg	Pred	MMF	5 years

M, male; F, female; ARR, Annual recurrence rate; Pred, P rednisone ;RTX, rituximab; MMF, mycophenolate mofetil.

### Treatment outcome

The average follow-up period was 3.8 years (range: 2–5). Of the seven patients with a typical relapsing-remitting course, three (42.9%, 3/7) failed with immunosuppressants but succeeded in controlling relapse with RTX. Four patients (patient 1 to patient 4) added RTX to adjuvant therapy. Patients 1, 2 and 3 still could not control symptoms even though tacrolimus was added for more than three months, so we changed tacrolimus to RTX, and then all of the patients stopped prednisone successfully and lesions shown by MRI became smaller or disappeared gradually.

**Figure 2:**
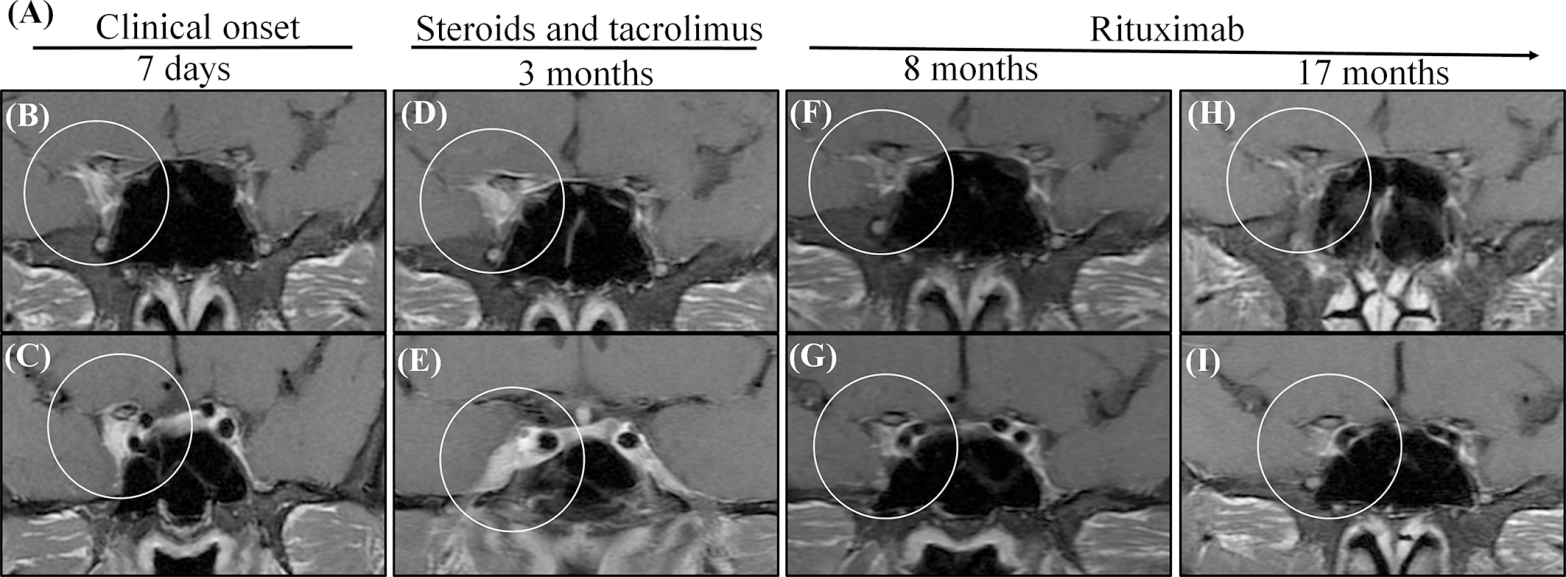
MRI shows that administration with steroids and tacrolimus was not successful for patient 1, while administration with RTX. **(A)** The summarized clinical course and therapy of the patient. **(B, C)** Initial MRIs showed a heterogeneous contrast-enhanced mass in the right cavernous sinus (circle). **(D, E)** Two times consecutively, the symptoms relapsed when prednisone was reduced to 40 mg per day although he oral tacrolimus at the same time. The MRIs obtained after 3 months reveal an enlarged size and enhancement of the cavernous sinus; the dura was involved [circle in **(E)**]. **(F, G)** After changing tacrolimus to RTX 5 months, in the MRIs obtained 8 months after the onset, the cavernous lesion disappeared (circle). **(H, I)** After stopping prednisone for 1 year and RTX for half a year, there was still no recurrence 17 months after the onset, and there were still no lesions in the cavernous lesion.

Lesions in the cavernous sinus of patient 1 totally disappeared when the tacrolimus was replaced by RTX at 5 months; when RTX was discontinued at 1 year, the disease remained stable for the following year (**Figures 2A–I**). After using RTX for two months, this patient had a pulmonary infection. In the fifth month of using RTX, he had hypoimmunoglobulinemia and his symptoms improved after symptomatic treatment. We can assume that pulmonary infection and hypoimmunoglobulinemia are side events of RTX. The lesion size in patient 2 is relatively large; she has been treated with RTX for two years with no relapse, but the lesion is still present. The lesions in patient 3 disappeared after treating with RTX for one year, so we stopped RTX; no recurrence occurred while following up for three years. Patient 4 had vision loss that deteriorated to no light perception of the right eye within two months; intravenous methylprednisolone 500 mg per day for five days did not restore vision acuity. Because the optic nerve, orbital apex and cavernous sinus were all involved and vision function was severely impaired, mycophenolate mofetil was added. Unfortunately, the other eye had vision loss for an interval of eight months, so we changed mycophenolate mofetil to RTX and kept the lesion in a stable condition. The lesions disappeared in the second year of treatment with RTX.

**Figure 3:**
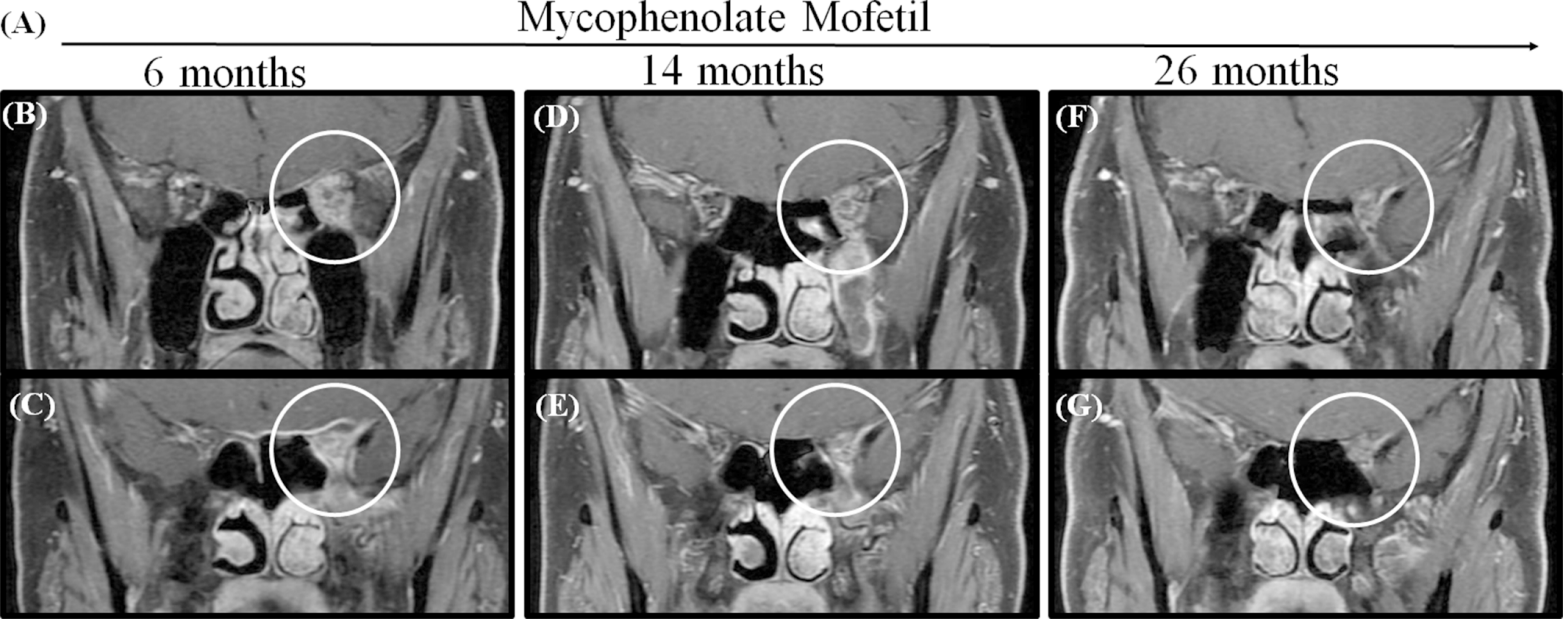
MRI shows that the lesion became gradually smaller in patient 9 when administrated with Mycophenolate Mofetil for more than 2 years. **(A)** The time axis of used Mycophenolate Mofetil. **(B, C)** MRIs obtained after using Mycophenolate Mofetil for 6 months showed a contrast-enhanced mass extending the orbital apex to the cavernous sinus (circle). **(D, E)** The lesion reduced in 14 months. **(F, G)** The lesion got smaller in 26 months but did not disappear.

Seven patients were treated with immunosuppressants. Four of these patients (patient 5 to patient 8) also received tacrolimus, and three (patient 9 to patient 11) also received mycophenolate mofetil as adjuvant therapy. Four patients (57.1%, 4/7) who had a typical relapsing-remitting course were able to undergo successful management with immunosuppressants. We suggested that patients stop immunosuppressants until the lesions disappeared. Then, all patients were treated with immunosuppressants for more than two years. This is longer than the time patients were treated with RTX. For patient 9, who had the lesion involvement of the optic nerve and whose lesion extended to the left orbital apex and cavernous sinus, vision acuity reduced to no light perception and he was managed with mycophenolate mofetil for more than three years. This caused the lesion to reduce gradually but did not make it disappear, and to this day, vision acuity has not been restored in this patient (**Figures 3A–G**).

## Discussion

The exact etiology of OIP is unknown, although abnormal immune response activation and infection have been suggested (6, 17, 18). More recently, a T-cell-mediated process in the pathogenesis of OIP has attracted attention (5, 19). Histopathologically, a mixed cellular inflammatory response is the rule, including lymphocytes (predominantly T cells), plasma cells, and neutrophil and eosinophil granulocytes accompanied by varying degrees of fibrous connective tissue hyperplasia (16, 20). These pathological findings give us a theoretical basis for treatment choice, including corticosteroids, immunosuppressants and B-cell depletion agent RTX.

Typically, OIP can affect any soft tissue component. When the disease affects the optic nerve sheath and posterior orbit and involves intracranial extension, it may also present with optic perineuritis, orbital apex syndrome and cavernous sinus syndrome, which may produce signs of optic neuropathy accompanying ocular motor disturbances that resemble multiple central nervous alsies and proptosis. A study found that one patient with OIP extracranial extension remained blind after 14 months of treatment (21), while other studies did not observe such severe vision loss (2–4). It is worth noting that 63.6% of the patients in our study had decreased vision at onset of disease; among them, 57.1% had severely impaired vision with little to no light perception. This is a reminder that rapid progression and complete loss of vision may occur in OIP with optic perineuritis and orbital apex syndrome. It can still be concluded that OIP has an unfavorable prognosis when the optic nerve is involved. It needs timely and effective treatment.

Seven patients (63.6%) in our study had a relapse course when prednisone was reduced to 15–40 mg or discontinued. A total of 57.1% were able to undergo successful management when this treatment was combined with immunosuppressants. The efficacy of combining prednisone with immunosuppressants is worse than in previous studies, which indicating that approximately 78% of patients treated with corticosteroids alone would have a relapse course, while this proportion decreases to 16% if combining with immunosuppressants (7, 8). The treatment with corticosteroids combined with immunosuppressants that patients received was unsatisfactory and so more effective treatment methods need to be found.

In this cohort, three of seven patients (42.9%) failed to control relapse with tacrolimus and mycophenolate mofetil, but after replacing immunosuppressants with RTX, they showed a dramatic improvement of signs and symptoms and lesions were obviously reduced or even completely disappeared. In a dose-randomized clinical trial of RTX therapy for refractory orbital inflammation, ten patients with orbital inflammation refractory to systemic corticosteroids and at least one other immunosuppressive agent were enrolled. Five patients were diagnosed with idiopathic OIP. Seven patients (70%) demonstrated improvement of symptoms with RTX (9). A review draws the conclusion that RTX appears to be both an efficacious and well-tolerated therapy for patients with non-infectious/non-malignant orbital inflammation (15). We can assume that RTX has a better efficacy compared to immunosuppressants in highly relapsing or refractory orbital inflammatory pseudotumors with intracranial extension.

In a clinical trial of RTX therapy for refractory orbital inflammation, the efficacy of RTX doses of 500 mg and 1000 mg in the treatment of orbital inflammation were compared and no difference in efficacy, duration of effect, B-cell depletion, or toxicity between the two dosages was found (9). A study showed that a dose of 100 mg RTX is effective in patients with active moderate to severe Graves’ orbitopathy (22). We applied 200 mg RTX to treat OIP with intracranial extension; the symptoms of all patients were controlled well with this dosage. Therefore, low-dose RTX can also be effective in patients with active refractory orbital inflammatory pseudotumors with intracranial extension.

RTX and immunosuppressants are well tolerated and generally safe (23–25). However, side effects are also noteworthy. Patient 1 had pulmonary infection and hypoimmunoglobulinemia after using RTX for two months. Other patients did not show severe adverse events.

A few limitations exist in this cohort study. The small sample size was an obstacle in the evaluation of the use of RTX in long disease courses. Another limitation was that biopsies were not feasible in most cases because the affected tissue was not readily accessible (e.g., orbital apex or cavernous sinus), although two patients with severe and irreversible vision loss performed biopsies to refute other confounding diagnoses. Prospective studies with a larger sample size are warranted to further concern the biological therapy in those patients.

## Conclusions

OIP extending to intracranial is uncommon. More than half of patients with this condition may be satisfactorily treated with corticosteroids combined with immunosuppressants. However, for patients who still experience recurrence or slow reduction of lesions after applying this combined therapy, RTX may be a better option.

## Data Availability

The original contributions presented in the study are included in the article/supplementary material. Further inquiries can be directed to the corresponding authors.
